# A multi-level framework for the functions of emotions and their expressions

**DOI:** 10.1098/rstb.2025.0150

**Published:** 2026-09-24

**Authors:** Laith Al-Shawaf, David M. G. Lewis, Elias Acevedo

**Affiliations:** ^1^ Department of Psychology, University of Colorado, Colorado Springs, CO 80918, USA; ^2^ Department of Biology, University of Colorado, Colorado Springs, CO 80918, USA; ^3^ Lyda Hill Institute for Human Resilience, University of Colorado, Colorado Springs, CO 80918, USA; ^4^ Murdoch University, Perth, WA 6150, Australia; ^5^ University of California Los Angeles, Los Angeles, CA 90034, USA

**Keywords:** emotion, emotion expression, levels of analysis, coordinating mechanism, proximate and ultimate, function, context, facial expressions

## Abstract

This paper introduces a framework for understanding the functions of emotion and emotion expression at multiple levels of analysis. The framework clarifies several key but sometimes overlooked conceptual distinctions in emotion science, including the distinctions between emotions and their expressions, the individual and social functions of emotions, and functions and benefits. This framework also integrates ultimate and proximate levels of analysis by showing how functional hypotheses lead to concrete, testable proximate predictions about how emotions work. By organizing these distinctions and levels of analysis, this framework provides a basis for asking precise questions and testing new hypotheses about the structure and function of emotions and their expressions.

This article is part of the theme issue ‘Mechanisms, development, phylogeny and functions of emotional expressions’.

## 1. Introduction

What are emotions, and how do they work? A central goal of emotion science is to elucidate how emotions operate and the functions they serve. However, achieving this goal depends on distinguishing clearly between different levels of analysis. In this paper, we advance a multilevel framework for understanding function in emotion and emotion expression across levels of analysis that vary in abstraction and specificity. This framework highlights several key conceptual distinctions—between emotions and emotion expressions, social and individual functions, and functions and benefits—and shows how functional hypotheses lead to concrete, testable predictions about how emotions work at the proximate level. We illustrate how this framework can be used to ask more precise questions in emotion science and to guide empirical research on the structure and function of emotions and their expressions.

## 2. What are emotions, and how do they work? Emotions as systems that coordinate cognition, physiology and behaviour

One fruitful way to think about emotions is as ‘modes of operation’ or ‘coordinating mechanisms’: superordinate systems that orchestrate the activity of different elements of cognition, physiology and behaviour in service of solving an adaptive challenge [1–4]. For example, disgust protects us from disease, fear facilitates our escape from imminent danger, guilt motivates us to repair valued relationships, and pride helps us showcase our successes [5–7]. In fulfilling its respective function, each emotion coordinates a wide variety of bodily and mental systems—including attention, perception, memory, learning, conceptual categorization, procedures for drawing inferences, physiology and behaviour—that facilitate that emotion's problem-solving function [1,4]. As a case in point, fear hones attention on potential threats (a psychological change), shunts energy from the digestive system to the skeletal muscles (a physiological change) and prompts defence or escape (a behavioural change). These changes, along with others prompted by fear, are functionally coordinated to help the organism avoid harm [4,6].

### (a) The flexible, context-sensitive design of emotions

A functional, evolutionary perspective does not imply that emotions will be rigid or that their activation will unfold in an automatic and stereotyped manner [1,3,8–11]. Evolved systems, including emotions, are sensitive to context [12–16] and would not be remotely functional if they were unresponsive to social, cultural and ecological conditions (e.g. [17]). For example, in line with *a priori* predictions generated by an evolutionary perspective, studies show that disgust is adaptively suppressed or downregulated towards one's offspring [18], and that anger and punitive sentiment are lower when the perpetrator of a crime is a genetic relative but higher when the perpetrator has harmed one's genetic relative [19], and attentional adhesion to attractive potential mates is strongly attenuated when one is in love [20]. An evolutionary approach emphasizes emotions’ sensitivity, flexibility and responsiveness to context [9,10].

The core idea of the coordinating mechanisms perspective, then, is that emotions are evolved adaptations that register a problem, take stock of its context and flexibly coordinate the activity of a variety of psychological and physiological systems in service of solving that problem. On this view, each emotion (or emotion family) is geared towards solving a particular adaptive problem (or family of problems). It should, therefore, be possible to develop a working taxonomy of emotions and their putative functions. Such a preliminary taxonomy is offered in table 1 — but we stress that emotion science is young, that the proposed taxonomy is tentative and subject to further confirmation and that many open empirical questions remain in the study of emotions from an evolutionary perspective (see [21]).

**Table 1.** A preliminary working taxonomy of emotions and their hypothesized functions.

emotion	hypothesized function
disgust	*pathogen disgust:* avoid pathogens, reduce the likelihood of infection *sexual disgust:* avoid inappropriate mating partners, avoid injudicious sexual and romantic choices *moral disgust:* coordinate group responses to offence, coordinate condemnation of the person and the offence, promote avoidance or punishment of that person
fear	escape or avoid imminent danger
sadness	withdraw, conserve energy, decide if major life changes need to be made or if effort needs to be allocated differently
grief	cope with the loss of a loved one, analyse possible mistakes or alternative paths, recalibrate in order to avoid future losses
regret	recalibrate decision-making procedures or parameters to improve future decisions
schadenfreude	facilitate the downfall of rivals or tall poppies by endorsing, working towards and/or refraining from obstructing it
anger	negotiate/demand better treatment and/or deter exploitation
jealousy	defend against threats to a valued relationship (often but not exclusively romantic)
envy	motivate the acquisition of what one currently lacks, increase one's relative standing in the social hierarchy, decrease another's standing
hatred	sabotage or eliminate rivals
contempt	signal that the target of contempt is of low status and/or motivate avoidance of the target
embarrassment	avoid status loss; apologize for social missteps; reassure others that one is aware of the norms, that the violation was accidental and that it won't happen again
guilt	repair a relationship in which one has not sufficiently valued or attended to an important social partner, alter future behaviour towards that person
shame	motivate avoidance of status loss, conceal information that might lead to status loss, repair and apologize (mitigate damage) if status loss or ostracism has occurred
pride	motivate achievement, showcase achievements in order to rise in social status
love	*romantic love*: pair bond, commit to one's partner, remain present to care for offspring *parental love*: raise offspring, protect offspring, provide offspring with tools for success
compassion	motivate helping and caregiving behaviours in response to others’ suffering or unmet need
empathy	facilitate understanding of others’ internal states and motivate compassion or other responses (e.g. distress)
gratitude	increase the value placed on a social other
happiness/joy	motivate the pursuit (and redeployment) of behaviours that promote fitness
surprise	recalibrate expectations about the world or specific stimuli in it
curiosity	motivate acquisition of new information, learn about one's environment, acquire new skills
boredom	motivate exploration and reallocation of attention to more rewarding pursuits
awe	process and harvest highly fitness-enhancing information; revise one's cognitive model of the world
lassitude (the ‘sickness emotion’)	conserve energy, adjust rest and eating behaviour, allocate energy to immune function, prevent further injury and illness
lust or sexual arousal	obtain and retain mate, motivate sexual congress
pain	avoid (further) tissue damage, disengage from pain-inducing task or stimulus
hunger	acquire and consume food to maintain homeostasis and promote survival

*Notes*. 1. This list is intended to organize existing functional hypotheses and illustrate that such a taxonomy is possible and represents a worthwhile goal. It is not meant to be definitive. 2. Emotions are ordered roughly by group, with ‘negative’ emotions listed before ‘positive’ emotions, and epistemic emotions listed together. 3. Some of the states listed here (e.g. hunger and pain) are not conventionally described as emotions but are included because they meet the criteria of a coordinating mechanism: they coordinate cognition, physiology and behaviour in response to adaptive challenges.

## 3. The functions of emotions—and how to test evolutionary–functional hypotheses

Table 1 lists the proposed evolved functions of a variety of emotions, ranging from anger to romantic love to lassitude. These proposals vary in the amount of empirical evidence accrued to date. Some of these emotions are relatively well studied, such as fear, disgust and anger [6,22–26]. Others have received a modest amount of attention, such as sadness, joy and envy [27–29], but much remains to be done. There are others whose evolved functions we're only just beginning to understand, such as lassitude, awe and boredom [30–32].

A central question in emotions research is how to appropriately test hypotheses about the evolved functions of emotions. A recurrent critique alleges that evolutionary hypotheses are ‘just-so stories’ [8,33–36]. According to this idea, because behaviour doesn't fossilize and because we don't have a time machine to travel into the past, evolutionary hypotheses about behaviour are, therefore, untestable (e.g. [37]). There is, however, a systematic way to test evolutionary hypotheses—and to ensure that one does not inadvertently fall into just-so storytelling (i.e. *post hoc* explanation without empirically testing the explanation). We describe one key element of this systematic approach below (for a more complete discussion, we refer interested readers to the systematic approaches described in [8,34,35,38–40]).

### (a) Ultimate (functional) hypotheses lead to proximate predictions

A key but under-appreciated point about testing evolutionary hypotheses that we wish to emphasize here is that researchers test functional (evolutionary) hypotheses via the mechanistic predictions that these hypotheses yield (see extended discussion in [34]). To phrase it differently, functional evolutionary hypotheses are typically located at the ‘ultimate’ level of analysis. They take the form ‘the adaptive function of anger is X’ or ‘the adaptive function of disgust is Y’. These ultimate-level hypotheses yield predictions at the ‘proximate’ level of analysis. Typically, these proximate-level predictions are concrete mechanistic predictions about how the emotion ought to work if the proposed function is correct [34].

For example, if one evolved function of disgust is to avoid pathogen threats, this leads to a variety of predictions, including that the emotion of disgust will be cross-cultural; some (but not all) substances will be universally disgusting; disgust will be triggered more strongly by pathogenic substances than by non-pathogenic substances (and will scale with the magnitude of a pathogen threat); disgust will trigger avoidance behaviour; the operation of disgust will be context-sensitive, such that the emotion is downregulated or suppressed when the pathogen source is one's offspring or kin; activating disgust will make people temporarily less gregarious and less open to experience; and the emotion of disgust will be characterized by a better-safe-than-sorry error management bias. All of these predictions have been empirically verified in subsequent tests [18,23,34,41–45]. This underscores the key point: specifying what the emotion is for (why it evolved) generates precise predictions about the operating characteristics of the emotion if the hypothesis about that emotion's function is correct.

Of course, a given adaptive function may be realized by multiple alternative designs, which means there is rarely a perfect one-to-one mapping between a function and its proximate instantiation (e.g. [34]). Nonetheless, functional hypotheses sharply constrain the solution space of viable mechanisms by ruling out the vast majority of designs that would fail to solve the adaptive problem at hand and by guiding researchers towards those designs or features that might succeed. Hypotheses about a system's function naturally lead to concrete *a priori* predictions about how the system will work.

### (b) The correspondence between form and function—functional hypotheses constrain form

In biology, a familiar way of putting this is that there is a tight fit between ‘form’ and ‘function’ (e.g. [46,47]). In a discipline that studies tangible physical structures like anatomy or physiology, ‘form’ refers to the physical form of the bodily structures under investigation. By contrast, in psychology and cognitive science, ‘form’ refers to the information-processing structure of the proposed cognitive system: what inputs it takes, how it processes those inputs and what outputs it yields [34]. One can use hypotheses about an emotion's function (what problem it evolved to help solve) to generate specific predictions about its form (its operating characteristics or how it works—for example, what inputs trigger its activation, what representations and algorithms it employs, what outputs it generates in response to specific triggers and what inputs lead to its deactivation [48,49]). These predictions about ‘form’ can then be readily tested in psychological studies.

The predictions that functional hypotheses yield are not only testable—they are novel. Consider shame, for instance. Traditional psychological accounts propose that shame is primarily elicited by negative self-evaluation, such as when one attributes a personal failure or wrongdoing to stable aspects of oneself (e.g. [50]). By contrast, a functional evolutionary hypothesis suggests that shame manages threats of social devaluation (i.e. negative evaluations of oneself by *others*), which leads to a different proximate prediction: shame should be calibrated to the anticipated evaluations of others, not to negative self-assessment *per se*. Empirical work supports this prediction. Across cultures, shame intensity closely tracks the degree of devaluation that an individual expects from others, even when no wrongdoing has occurred [51]. In addition, the assessments of others substantially outperform self-assessments in predicting the degree of shame a person feels [52].

In sum, hypotheses about *why* an emotion exists (what it is ‘for’) yield predictions about *how* the emotion works. These predictions can be readily tested with the psychological researcher's standard toolkit: behavioural experiments, cognitive experiments, surveys, observational studies, neuroimaging, psychophysiology and so on [8,53]. A key takeaway is that testing hypotheses about the evolved functions of emotions does not require a time machine to travel into the past. Because evolutionary-functional hypotheses furnish predictions about how the emotion is expected to work *in the present day*, testing them does not require any special tools; it merely requires researchers to use an evolutionary hypothesis to generate proximate, present-day predictions and then test them.

## 4. Facial expressions of emotion: important, but not an evidentiary criterion for adaptation

Historically, a major area of research in emotion science has centred on the expression of emotions, often in the face [41,54,55]. Some—but, crucially, not all—emotions have an accompanying facial expression. Some classical examples of those that do include fear, anger, surprise, joy, sadness and disgust [55,56]. Some examples of emotions that appear not to have a unique, diagnostic facial expression—and whose recognition may depend on contextual clues—include jealousy, envy, regret and schadenfreude [41,57–59]. There are also emotions that include a bodily expression but not a facial one (e.g. pride and shame) and thus were often excluded from earlier historical analyses that focused exclusively on the face [60,61].

In this line of research, an influential historical view held that an emotion should only be considered evolved if it comes along with a facial expression that is both universal and universally recognizable [41,56]. However, more modern approaches to emotion science suggest that this stipulation is unnecessary and misleading [1,12,13]. Whether an emotion is accompanied by an expression depends on the (ancestral) net costs and benefits of displaying the emotion to others. There are many contexts in which it is beneficial to signal emotions to conspecifics—for example, signalling to one's offspring fear of a dangerous animal or disgust towards pathogenic substances. However, there are also many contexts in which it is not beneficial to signal one's affective state, such as when one is experiencing envy, jealousy or shame about a transgression of which no one else is aware. This means that some emotions may come with a universal facial expression, but others may come with no expression at all —and even those that have a universal facial expression may display that expression in a context-sensitive manner. Emotions may also come with a bodily expression but not a facial expression.

In short, there is no theoretically grounded, non-arbitrary reason to insist that emotions must have facial expressions, or that these facial expressions must be universal (for further discussion, see [12,62]). Instead of this disproportionate focus on facial expressions as an evidentiary criterion for establishing that an emotion has an evolutionary basis, researchers can make progress by analysing each emotion on a case-by-case basis to investigate which emotions include signalling as part of their evolved function, which do not, and—of those that do—when (i.e. in what contexts) signalling the emotion is beneficial, and when it is not [62].

The above analysis has implications for what kinds of conclusions we can or cannot draw based on the presence or absence of facial expressions of emotion. The key evidentiary criterion in deciding whether or not an emotion is an adaptation with an evolved function is not whether it has a facial expression, but rather whether it exhibits ‘special design’ [63]: does it appear well engineered to solve the adaptive problem that it is hypothesized to have evolved to solve? Does it exhibit a tight *form-to-function fit* (is it highly improbable that the correspondence between problem and solution could have arisen by chance)? If so, it may be an evolved adaptation whose function is to solve that problem [14,63–66]. Facial expressions are interesting and important for other reasons, but their presence or absence is only relevant to this question to the extent that it helps answer these questions about special design.

As researchers, if we are interested in testing whether an emotion is an evolved adaptation, we can focus our efforts on (i) identifying or developing a clear *a priori* hypothesis about the evolved function of the emotion, (ii) generating specific proximate predictions that flow from this hypothesis (if the emotion indeed evolved for this function, then what inputs should it process? What outputs should it produce? In what contexts should we expect the emotion to be signalled to others versus not?) and (iii) testing these predictions in empirical research. This systematic approach to generating and testing functional hypotheses has yielded new predictions and discoveries about a wide variety of emotions [21] and promises to continue to do so as emotion science grows.

## 5. The multiple functions of emotion expression

### (a) The function of emotion expression at the broadest level: signalling to others

Throughout this article, we use the term ‘function’ at multiple levels of abstraction (see table 2 for a detailed schematic). At the broadest level (Level 1), the function of adaptations—including emotion expression^1^ —is to enhance an organism's inclusive fitness by promoting its survival and reproduction and/or those of its relatives (which share its genes) and allies (who reciprocally promote the organism's own survival and reproduction).

**Table 2.** Functions of emotion expression across levels of abstraction and specificity.

level of abstraction	what ‘function’ means	scope	illustration	need for additional specificity beyond this level (what remains to be explained)
1. evolved adaptations in general (broadest level)	what adaptations are for	all adaptations (not just emotion expression)	‘The function of adaptations is to enhance the organism's inclusive fitness.’	‘Ok, but how do emotion expressions contribute to achieving this function?’
2. emotion expression in general	what emotion expressions are for	all emotion expressions, regardless of which emotion	‘The function of emotion expression is to communicate the state of oneself or the world to others.’	‘Ok, but what exactly does expressing a specific emotion like disgust communicate?’
3. the expression of a specific emotion	what *this* emotion's expression is for	the expression of a particular emotion	‘The function of the disgust expression is to warn others about pathogen hazards.’	‘Ok, but why does the disgust expression take the specific form that it does?’
4. the mechanistic implementation or form of the emotion expression	what the *form* of the expression is for	the anatomical or behavioural actions that instantiate the expression	‘The function of nasal wrinkling when expressing disgust is to reduce airborne pathogen intake.’	‘Ok—and why is the disgust expression more pronounced in certain contexts, such as when interacting with children?’
5. context-specific deployment of this emotion expression (most specific level)	what the expression is for *in this particular context*	the expression of a particular emotion *in a particular context or situation*	‘Adults may exaggerate the disgust expression to teach children local avoidance norms.’	*—*

However, this function is so broad that it applies to all evolved adaptations, not just emotions or emotion expressions. How, then, does emotion expression specifically enhance fitness? More precise analyses of function answer this question by identifying what emotion expression is *for* with increasing granularity or precision. This can be done for emotion expression in general (Level 2), for a specific emotion's expression (Level 3), for the design features of a specific emotion's expression (Level 4) and finally for how the deployment of a specific emotion expression responds to context (Level 5).

At a relatively broad level (Level 2 in table 2), a central function of emotion expression is to communicate the emotion (and, in doing so, the state of the world, such as a threat or opportunity) to others. In principle, this function applies to any emotion that involves signalling.

At a more specific level (Level 3), the function of expressing a given emotion depends on that emotion. For example, the fear expression signals to one's peers that danger is close, the disgust face alerts friends and family to the presence of dangerous pathogens or to moral violations, and the joy smile may reassure others that one is happy and there is no present danger [28,69,70]. The anger face communicates to others that they have not treated one well enough, that the angry person demands better treatment and that they may be preparing to impose costs or withdraw benefits to accomplish this goal [71]. Expressing pride lets one's peers know about an accomplishment, and advertising this success is often beneficial in gaining respect or climbing the social hierarchy [72]. After one is observed engaging in an unwanted or undesirable behaviour, signalling shame usually conveys that one acknowledges wrongdoing, is willing to appease other members of the social group and accepts subordination [7].

### (b) The function of expressing a specific emotion depends on the emotion and the context

Each of the above examples falls under the broader umbrella of signalling to others in one's social group. However, each expression has a more specific function—soliciting aid, apologizing for wrongdoing, advertising success and so on—that must be understood not only in relation to the emotion being expressed but also in relation to the context in which it is being expressed.

This is a crucial point because the function of expressing a specific emotion can vary across contexts. For example, expressing disgust or fear has a different function when used to warn other adult conspecifics that are familiar with an environmental threat but have not yet detected its presence versus when parents make—or intentionally exaggerate—a disgust or fear display in front of their children in order to teach their kids what constitutes an appropriate object of disgust or fear in their local ecology and culture [12,73]. One might also attempt to suppress an expression of disgust or fear in order to signal tolerance of risk, formidability, or to dissuade others from attacking.

This means that while it is possible to understand the function of emotion expression for each emotion and for emotions in general, a more fine-grained analysis that incorporates context is called for when analysing the function of emotion expression at a more precise level. From a functional perspective, the relevant question is often not ‘what is the function of this emotion expression?’ in the singular, but rather ‘what functions does this emotion expression serve under which conditions?’ This approach allows researchers to generate and test predictions about expression deployment and context sensitivity without assuming a one-to-one mapping between expressions and functions that is uniform across all contexts (Level 5).

### (c) The anatomy and shape of emotional expressions are meaningful rather than arbitrary

Why do specific expressions correspond to specific emotions? In theory, we could imagine any emotion being expressed using any set of body postures, limb movements and facial muscle activations. That is, as long as the muscle and limb movements were consistent for a given emotion, the mapping could be arbitrary and still reliably convey that specific emotion. But by and large, this is not how emotion expression actually works—emotional expressions are not an arbitrary set of symbols that convey meaning by convention only (e.g. [74], 2.249). Rather, specific emotions are expressed in specific ways that either follow a functional pattern, reflect their phylogenetic ancestry or both [54].

For example, the anger face is not arbitrary: the lowered brow and flared nostrils specifically serve to make the signaller appear more imposing and formidable [71]. Pride expressions involve bodily expansion, increasing the apparent size of the expressor and signalling their success, competence or high standing. This leads to increased respect and social advantages. Shame expressions, by contrast, involve bodily contraction and a downward gaze, often partially obscuring the face, which function to signal submission or appeasement following a social transgression [7,75]. These non-arbitrary movements fit their social functions (rising and falling/accepting subordination in the social hierarchy, respectively).

Auditory expressions follow a similar logic. Human screams exhibit acoustic roughness—characterized by rapid temporal amplitude modulations in the 30–150 Hz range—a design feature that captures attention and triggers a fear response in listeners [76]. Here again, the acoustic form of the signal is tightly coupled to one of its hypothesized functions: alerting conspecifics to danger. The same is true of infant cries: they are characterized by non-arbitrary acoustic features such as high pitch and temporal irregularity that trigger affective systems associated with alarm and distress, are cross-culturally recognizable and successfully compel attention and caregiving behaviour [47]. The key point is that, for many emotions that do have an accompanying expression, expression is often not arbitrary—it often has its roots in, and is closely tied to, the function of the emotion.

In addition to their communicative functions, many emotion expressions also serve physiological or perceptual functions for the expresser. For example, the widened eyes, characteristic of fear, increase peripheral vision and sensory intake, facilitating rapid detection of potential threats [54,77]. The scrunched-up nose and pursed mouth associated with disgust reduce airflow through the nose and mouth, thereby reducing the likelihood of ingesting pathogens [77]. Disgust can also prompt a protruded tongue, which helps to expel pathogens that have already entered the mouth [78]. In short, the specific muscular activations associated with expressing fear and disgust are directly tied to their respective functions.^2^ The form of emotion expressions is thus often meaningful rather than arbitrary.

### (d) Whom do emotion expressions benefit: the self or others?

The individual and social benefits of emotion expression resist clean separation. The reason for this fuzzy boundary lies in the logic of natural selection, which operates more strongly at the level of genes than at the level of individuals. Much of the time, what benefits an individual also benefits their genes—if you stay alive and reproduce, so do your genes. However, the ‘interests’ of genes also extend beyond the individual bodies that they reside in, because organisms are genetically related to each other to varying degrees. Genes can, therefore, increase their frequency in a population when they preferentially benefit close genetic relatives who are more likely to carry copies of them, not just when they benefit the specific body they are in—a logic known as *inclusive fitness* [79]. This means that an emotion expression that imposes costs on the expressing organism can still evolve if it helps that organism's kin to survive and reproduce. The alarm calls of Belding's ground squirrels (*Urocitellus beldingi*) provide a classic example from the animal behaviour literature. Squirrels that sound an alarm run an increased risk of being caught by a predator, thereby incurring a personal cost, but they benefit their nearby kin by alerting them to the presence of the threat, which leads to a net benefit overall [80]. This causes the squirrels to sound the alarm despite the personal cost they incur in doing so.

Similarly, helping a friend in need may benefit oneself in the long run if that friend reciprocates down the line. Here, helping others can be favoured by selection precisely because doing so ultimately enhances one's own genetic reproduction—a logic known as *reciprocal altruism* ([81]; note that no conscious cost–benefit calculation is implied). Often, both organisms end up better off than they would have been without the exchange, a phenomenon known as ‘gains from trade’ [82–84].

These considerations highlight that the genetic interests of an organism often include the genetic interests of other individuals, which necessarily blurs the boundary between the individual and social functions of emotion expression. In fact, the individual benefits of expressing an emotion (the benefits to the expressor) are often realized through their beneficial effects on others. This means that individual and social benefits of emotion expression may often be inherently intertwined. In other words, although analytically distinct, these functions are often intertwined in practice.

Nonetheless, the individual vs. social distinction may be a useful conceptual tool for generating testable predictions at the proximate level. Mechanistic features of emotion expressions that primarily serve individual functions, such as the reduced airflow produced by the disgust face, may be expected to operate more independently of audience presence, whereas we should expect features of emotion expression that primarily serve social functions to exhibit greater sensitivity to audience-related cues. The expressive components of shame or embarrassment, for example, should be more likely to emerge or intensify when others become aware of a perceived transgression or norm violation.

#### (i) Mapping the distinction between individual and social functions onto emotions and emotion expressions

Emotions frequently serve a beneficial function for the individual even in the complete absence of any expression and even when the presence of an emotion's expression can be thought of as serving a social function. For example, the function of fear is to avoid or escape danger (individual), whereas expressing fear often functions to help others in the social group avoid or escape danger (social). Similarly, the function of disgust is to avoid pathogens and reduce the likelihood of infection (individual), but a major function of expressing disgust is to alert others in the social group to the presence of pathogens so that they can avoid infection (social).

Even when an emotion is inherently social, it often has a straightforwardly individual function: anger helps the individual secure better treatment, romantic love helps them form and maintain a beneficial pair bond, parental love helps them to invest in offspring that carry their genetic material into future generations, envy helps motivate them to do better in competitive endeavours and so on [25,26,29,85,86]. As noted, the individual function is often tied to a social function such that the two cannot be easily or cleanly disentangled from one another. In the case of disgust, for example, (i) experiencing the emotion has the individual function of avoiding pathogens; (ii) expressing the emotion has the social function of helping one's kin and allies to avoid pathogens; (iii) the specific form of the expression (the muscular contractions that constitute the disgust face) has the individual function of reducing the likelihood of ingesting pathogens; and finally, (iv) the social function of disgust—helping one's kin and allies to avoid pathogens—ultimately serves an individual function: because of inclusive fitness and reciprocal altruism, one benefits when one's allies and kin avoid harm. Thus, emotion expressions are often social in nature, and the social function is often tied to, or ultimately tributary to, an individual function.

#### (ii) Can emotion expressions have purely individual functions?

This analysis might prompt one to ask whether there are any purely individual functions of emotion expression. That is, does emotion expression have any functions that have nothing to do with communicating the emotion to others? Much less work exists on this question. Here, we discuss a few candidates for purely individual functions of emotion expression.

Experimental research shows that emotion expression can improve health outcomes (see [87] for a review of this literature). In the expressive writing paradigm, participants are randomly assigned to write for several brief sessions about deeply emotional or traumatic experiences or, in control conditions, about emotionally neutral topics. Across laboratory and clinical samples, participants assigned to emotional disclosure show subsequent improvements in mood, subjective well-being and objective health indicators—including immune functioning and other physiological markers (e.g. [88,89]). However, these may be benefits rather than true biological functions—a distinction that we cover in the next section.

A growing body of work also suggests that sighs function as a respiratory ‘reset’, helping to restore optimal breathing patterns and regulate physiological arousal [90,91]. Experimental and observational studies suggest that sighing facilitates recovery from stress [92]. These effects operate regardless of audience presence, which makes sighs a plausible example of an emotion-linked expression whose primary function is individual rather than communicative. More speculatively, these findings point to a functional hypothesis: sighing may be a homeostatic regulatory mechanism that shifts the organism from a state of heightened physiological arousal and affect—such as stress or anxiety—back towards baseline.

Another candidate might be crying, which some researchers have argued serves a self-soothing function [93]. If correct, this could be a candidate for an individual function of emotions that does not necessarily involve signalling to others (but see [94] for a different view).

A fourth candidate might be expressing an emotion out loud in words—this may serve the cognitive function of organizing and structuring one's thoughts, which may have downstream benefits on cognition or planning that do not necessarily involve signalling. Another possibility has to do with public commitment: expressing one's regret, gratitude or guilt may entrench the emotion more fully or may function to make one ‘go on the record’ as feeling a certain way or adopting a certain stance. Having publicly disclosed this emotion might subsequently change one's behaviour in beneficial ways that align with the publicly disclosed emotion. For example, after hurting a friend or loved one, a person might express guilt. Having expressed this guilt publicly, they might hold themselves to better behaviour in the future, reducing the likelihood of further damage to the relationship. In this way, public expressions of emotion may act as a ‘commitment device’ that shunts one towards a different behavioural path—one that holds individual benefits for the expresser in the future. This is a hypothetical idea that, to our knowledge, has not been tested yet. Of course, these expressions may also serve additional social functions. Public displays of guilt or regret, for instance, may also elicit forgiveness from one's social partners or help restore trust. Still, these examples illustrate how emotion expressions might hypothetically serve individual functions that are independent of their social signalling function.

While speculations about purely individual functions of emotion expression are not off the table, they do come with some caveats. First, they are understudied, and we know very little about them if they do exist. Second, for reasons outlined above, we emphasize that the distinction between individual and social will often resist clean separation when it comes to the function of emotion expression. Third, given the abundant social functions of emotion expression, if purely individual functions of emotion expression exist, they are likely to occupy only a small slice of the pie of overall functions of emotion expression.

### (e) The distinction between functions and benefits

This analysis of emotions and their expressions would not be complete without mention of another important distinction: the difference between functions and benefits. In biology, the function of a system, mechanism or trait is *why* it evolved: what problem it solved or what adaptive benefit (effect on survival or reproduction) played a key role in driving its evolution [2,3,63,66]. This is sometimes referred to as the trait's ‘proper function’ [65,95]. By contrast, a benefit is merely a positive effect conferred by the trait or system, without reference to whether it evolved for that specific reason. For example, a nose holds up eyeglasses—and while that is a definite benefit, it is not the evolved function of the nose. Just because something is currently adaptive or offers benefit X, this does not mean that the trait evolved *because of* benefit X [2,3,64,65]. Identifying a benefit offered by a trait is not the same as identifying that trait's proper evolved function [2].

The distinction is relevant in this context because it helps us parse the functions of emotion expression from the mere benefits of emotion expression. Generally, the function of an adaptation can be best understood at the genic and individual levels, rather than at the level of the larger group, society or species [63,79,96]. Applying this distinction to emotion expression, one might argue that while the benefits of emotion expression are often social, the function of emotion expression is, in a sense, individual. For example, expressing fear benefits group members because it enables them to detect and avoid danger, and expressing disgust likewise benefits group members because it helps them avoid pathogens. But, as discussed in §5d(i), the function of expressing these emotions can still be construed as individual in the sense that helping one's mate, offspring and allies to avoid danger ultimately benefits the individual expresser (and the expresser's genes)—and it is these benefits to the expresser that drove the evolution of the emotion's expression in the first place.

The distinction between benefits and functions can be mapped onto the prior distinction that we drew between the social and individual functions of emotions and their expressions. Consider a parent who, while walking with their child at a busy park, expresses disgust in response to dog faeces on the path ahead. In this case, the parent's disgust expression signals to the child what to avoid and also reduces airflow through the parent's nose and mouth, reducing the ingestion of pathogens. This display serves a social function that benefits the child. At the same time, the parent also benefits in multiple ways; the parent directly benefits from the reduced ingestion of pathogens and indirectly benefits from their child avoiding harm. These are individual functions. Strangers who happen to observe this exchange may also benefit, but this benefit is entirely incidental and does not count as either a social function or an individual function. The same disgust expression thus benefits all three parties, but only the benefits to the child and the parent correspond to the evolved functions of the expression. Note also that, as discussed earlier, the social and individual functions are causally intertwined: the expression's social function (signalling to, and thereby protecting, the child) simultaneously contributes to an individual function (protecting the child promotes the parent's inclusive fitness).

To offer a visual aid, table 3 summarizes the major conceptual distinctions drawn throughout this paper and indicates where in the paper each distinction is discussed in detail. These distinctions recur across different sections, and we hope researchers find them useful for thinking about emotions and emotion expression.

**Table 3.** Key conceptual distinctions in evolution, emotion and emotion expression.

distinction	what's being contrasted	core idea	why it matters	section(s) with further detail
emotion vs. emotion expression	emotion as a system vs. the emotion's outward expression	emotions are coordinating mechanisms. Expressions are signals they sometimes produce.	emotions often have a primary individual function. Expressions often have a primary social function (which itself contributes to an individual-level function).	§4; §5d(i)
ultimate vs. proximate levels of analysis	different types of explanation: ultimate = *why* it's like that (evolutionary, functional) proximate = *how* it works (often mechanistic)	ultimate hypotheses yield testable, concrete proximate predictions	both ultimate and proximate levels of analysis are necessary for a complete explanation crucial in generating hypotheses: we test ultimate ‘why’ hypotheses via their concrete proximate ‘how’ predictions	§3
function vs. benefit	selected-for effect (function) vs. other positive effects (benefit)	proper function = evolved for this reason benefit = can be incidental	anchors claims of adaptation in evidence of special design. Claims about evolved function require evidence of tight *form-to-function fit.*	§5e
individual (intra-personal) vs. social (interpersonal)	focused on the expresser vs. focused on others	many emotions have a primary individual function many emotion expressions aid others, yet simultaneously advance the expresser's interests	identifies who benefits from an emotion's expression. clarifies why social pay-offs often translate into individual pay-offs and why social and individual are so often intertwined.	§5d; §5d(i); §5d(ii); §5e
*function* can be profitably analysed at different levels of precision or granularity	broadest (Level 1) → most specific (Level 5)	adaptations → emotion expression in general → emotion-specific expression → form/mechanism of an emotion expression → emotion expression in specific contexts	helps locate claims along the causal chain helps frame different questions helps avoid over-/under-generalization that can result from inadvertently tackling the wrong level of specificity	§5a; §5b; §5c; table 2
genic-level vs. organism-level selection	genes’ ‘interests’ vs. an individual organism's interests (no conscious calculation implied for either)	owing to the logic of inclusive fitness, functions can extend beyond the individual organism to kin and allies	clarifies why the boundary between individual and social functions is inherently fuzzy (kin selection, reciprocity)	§5d; §5e

## 6. Summary and conclusion

In this paper, we've suggested that a productive and generative way of understanding emotions is as ‘coordinating mechanisms’: regulatory systems that coordinate a variety of programmes in the body and mind in service of solving an adaptive problem in a context-sensitive manner. On this view, each emotion has a function and evolved for a reason [1,3,4,97]. Consequently, it should be possible to develop a taxonomy of the evolved functions of known emotions. Table 1 lists a preliminary, non-comprehensive taxonomy of this kind as an illustration.

How can one test hypotheses about function? Contrary to a common misconception, testing hypotheses about why emotions evolved does not require a time machine. Functional hypotheses (i.e. ultimate-level or *why* hypotheses) furnish concrete predictions about how we should expect an emotion to operate (i.e. proximate or *how* predictions) if indeed the emotion evolved for the hypothesized function. These predictions about the operating characteristics of the emotion—what environmental inputs trigger its activation, how the emotion responds to context, and how the emotion processes cues and generates context-sensitive behavioural outputs—can then be tested using the standard methods and data sources that social scientists typically employ. In other words, hypotheses about the evolved functions of emotions are empirically testable.

Some of these testable predictions concern emotion expression, but as we emphasize in §4, expression is only one facet of emotion, and its presence or absence is not an appropriate evidentiary criterion for whether an emotion has evolved. Instead, the relevant evidentiary criterion is the degree of form-to-function fit: the degree to which the system's operating characteristics are well-suited to solving its hypothesized adaptive problem (e.g. [2,63]).

We suggest that the core concept of function can be analysed at different levels of specificity (table 2). At the broadest level, the function of emotion expression is to signal one's emotion to others. At a more precise and fine-grained level, the function of emotion expression depends on the specific emotion in question: for example, expressing fear communicates the presence of danger, whereas expressing sadness informs loved ones that one needs help and support. At an even more granular level, the function of emotion expression depends on context: for example, one might suppress a disgust response in order to appear brave in front of friends or a mate, whereas one might exaggerate a disgust expression in order to teach one's children the appropriate objects of disgust in one's local culture and ecology [12,73]. Similarly, depending on context, one might express fear to alert others of an imminent threat or to de-escalate conflict by signalling distress and submission, prompting reduced hostility from an opponent [98]. Table 2 lists these functions in detail, proceeding from the most general level of analysis to the most specific.

The form of an emotion's expression is often tied to a specific biological function. For example, the anger face makes one look more formidable, which enhances bargaining power [71], the disgust face serves to reduce the likelihood of pathogen intake via the nose and mouth (e.g. [77,78]), and the acoustic form of infant cries compels caregiver attention [47]. In such cases, the emotion expression—not just the emotion itself—exhibits a tight, non-arbitrary form-to-function fit.

To test the hypothesis that an emotion or emotion expression has an evolved function, researchers must demonstrate that the operating characteristics of the emotion (or expression) are structured and organized such that the emotion (or expression) is well-suited to solving the problem it is hypothesized to have evolved to solve [2]. Researchers can feel confident in concluding that they have correctly identified the proper function of the emotion under investigation only when this form–function fit is tight, and will benefit by actively refraining from drawing firm conclusions or making strong claims of adaptation when it is not.

In this paper, our goal has been to (i) integrate ultimate and proximate levels of analysis and identify the relationship between the two in empirical psychological studies, (ii) apply this distinction to emotions in a systematic way, (iii) disentangle functions from benefits in emotion science, (iv) distinguish between emotions and their expressions, and (v) define functions at different levels of specificity. We hope this paper provides a roadmap for asking more precise, granular questions about the design and operation of emotions and their expressions. We look forward to researchers building on this framework and exploring the many open questions that remain about the architecture and adaptive functions of emotions.

## Data Availability

This article has no additional data.

## Footnotes

^1^
We treat verbal and non-verbal expressions as sharing a common functional logic insofar as both serve as outputs of emotion systems that convey information to others. That said, it is worth noting that these expressive modalities can differ in their degree of voluntary control. Verbal expressions are often more easily decoupled from underlying affective states. Non-verbal expressions, by contrast, are often more tightly linked to underlying physiological and perceptual processes, which can make them harder to fake and, thus, more reliable signals (e.g. [67,68]).

^2^
The configuration of an expression may have initially evolved to regulate perception or physiology in the expresser and later became co-opted for communicative functions. Where such an evolutionary sequence has occurred, the function of the physical configuration of an emotion's expression can be analytically distinct from the functions of signalling that emotion to others. In such cases, the emotion expression may still serve the original physiological or perceptual function and serve the communicative function—these represent independent, testable hypotheses about the emotion expression.

## Theme

One contribution of 19 to a theme issue ‘Mechanisms, development, phylogeny and functions of emotional expressions’.
